# Cryogelation and Cryogels

**DOI:** 10.3390/gels5040046

**Published:** 2019-12-03

**Authors:** Zachary J. Rogers, Sidi A. Bencherif

**Affiliations:** 1 Department of Chemical Engineering, Northeastern University, Boston, MA 02115, USA; rogers.z@husky.neu.edu; 2 Department of Bioengineering, Northeastern University, Boston, MA 02115, USA; 3 Laboratory of Biomechanics & Bioengineering (BMBI), Sorbonne University, University of Technology of Compiègne (UTC), 60200 Compiègne, France; 4 Harvard John A. Paulson School of Engineering and Applied Sciences, Harvard University, Cambridge, MA 02138, USA

Cryogenic processes are increasingly being utilized to create unique polymeric materials that tackle challenges mainly in the biomedical arena, environmental science, and field of food technology. Cryogelation is a process in which gelation occurs under semi-frozen conditions, leading to a polymer network cross-linked around ice crystals [1,2]. Subsequent thawing of ice crystals leaves behind a polymeric material with an interconnected, macroporous network surrounded by a highly dense polymer wall. This material is commonly called a cryogel. In a biomedical setting, unlike conventional nanoporous hydrogels, cryogels allow unhindered diffusion of solutes of practically any size and promote infiltration, trafficking, and survival of mammalian cells [1,2]. When used as matrices for bioseparations, cryogels enable faster flow rates and subsequently faster separations when compared to conventional solid adsorbent materials [2]. Furthermore, the interconnected macropores and densely cross-linked polymer walls confer cryogels with exceptional elasticity and shape-memory properties, allowing them to be syringe injected through hypodermic needles [1,3]. Altogether, due to their unique properties, cryogels have been used for cell delivery, drug delivery, cancer immunotherapy, tissue engineering, bioseparations, biosensors, and cell culture in three dimensions [1]. On the other hand, cryostructurates, another type of cryotreated gels, are formed by a process in which gel formation occurs either before or after freezing [2]. As cryostructurates have not been studied as extensively as cryogels, more research is critically needed to expand their use.

In this Special Issue, “Cryogelation and Cryogels”, recent advances in the development, characterization, and application of cryogels and cryostructurates are described. In several studies, the impact of gel formation parameters on mechanical and physical properties were investigated. Bauldron and colleagues investigated the difference between freeze-drying and supercritical drying on the physical properties of amylomaize starch gels [4]. It was determined that freeze-drying resulted in macroporous gels with 20–100 µm pores, whereas supercritical drying led to gels with 1 µm pores. In another study, Muslomova and colleagues fabricated butyl rubber cryogels while varying the type of solvent, cryogelation temperature, and several other parameters [5]. Switching the type of solvent from benzene to cyclohexane decreased the pore size, whereas increasing the freezing temperature led to gels with more ordered and aligned pores. These types of studies allow for the fine-tuning of material properties to fit specific applications. Other studies looked in depth at specific parameters to understand their effects. For instance, Lozinsky and coworkers discovered that two chaotropic agents, which typically weaken the mechanical properties of cryogels when added to an aqueous cryogelation process, have the opposite effect on polyvinyl alcohol cryogels fabricated in DMSO (called “kosmotropic-like”) [6]. Focused more on applications, Hixon and colleagues compared electrospun scaffolds, hydrogels, and cryogels for their potential to be used as wound-healing, antimicrobial tissue-engineering scaffolds. However, based on their work, it appears clear that more in-depth studies are needed to determine which biomaterial is superior to achieve the best outcome [7]. 

Several review articles are included in this Special Issue to bring readers up to speed on the latest advances in the field. Saylan and Denizli review the biomedical applications of cryogels, with an emphasis on composite cryogels [8]. Distinctively, Kudaibergenov provides a deep dive into polyampholyte cryogels (i.e., cryogels made up of positively and negatively charged subunits), and their properties and applications as stimuli-responsive materials [9]. Lastly, Lozinsky distinguishes the difference between cryogels and cryostructurates, a much needed exercise as the field is growing rapidly [10].

Since the process of cryogelation was first implemented in the 1970s, scientists have made immense progress in the synthesis, characterization, and application of cryogels and cryostructurates. However, there is still room for improvement. Studies like that of Hixon and colleagues, in which multiple biomaterials are compared to determine the best fit for a specific application, are essential to advance the field [7]. For instance, in the biomedical field, there are several challenges that cryogels face, preventing their lab-to-clinic translatability, including sterility, degradability, and controlled encapsulation and release of drugs and/or biomolecules. Moving forward, the emphasis of future research should focus on in vivo studies that accurately recapitulate their real-world applications. We are excited as *Gels* publishes more research and applications that advance the state-of-the-art in the field, bringing us closer to cryogels’ use in everyday life. 
